# Relationship Between Occupational Safety and Health Policy Principles, Organizational Action on Work-related Stress and the Psychosocial Work Environment in Italy

**DOI:** 10.1016/j.shaw.2023.10.001

**Published:** 2023-10-05

**Authors:** Stavroula Leka, Luis Torres, Aditya Jain, Cristina Di Tecco, Simone Russo, Sergio Iavicoli

**Affiliations:** 1 Centre for Organizational Health & Well-being, Lancaster University, Lancaster, UK; 2 University of Nottingham, Jubilee Campus, UK; 3 Nottingham University Business School, Jubilee Campus, Nottingham, UK; 4 Department of Occupational and Environmental Medicine, Epidemiology and Hygiene, INAIL—Italian Workers' Compensation Authority, Rome, Italy

**Keywords:** ESENER, EWCS, Italy, OSH policy principles, Psychosocial risks

## Abstract

**Background:**

It is acknowledged that legislation acts as a motivator for organizational action on psychosocial risks. Our study aims to provide evidence on the relationship between key occupational safety and health (OSH) policy principles and organizational action on work-related stress, and, in turn, with reported employee job demands and resources and their experience of work-related stress. We focus on Italy where specific legislation and practices on work-related stress were introduced in 2008 which are underpinned by these key OSH policy principles.

**Methods:**

Secondary analysis of the Italian samples from the employer ESENER-2 and employee 6th EWCS surveys was conducted, using path analysis in structural equation modeling (SEM) linking the two datasets.

**Results:**

We found a strong statistically significant relationship between OSH policy principles and organizational action on work-related stress (C.I. = .62-.78 *p* < .001). The existence of an organizational action plan on work-related stress was found to be significantly associated with more reported job resources (C.I. = .02-.24, p < .05) but these were not found to be significantly associated with less work-related stress. No significant association was found between having an organizational action plan for work-related stress and reported job demands. However, job demands were significantly related to reported work-related stress (C.I. = .27-.47, p < .001).

**Conclusions:**

Findings add support to the call for specific legislation on work-related psychosocial risks and highlight how an organizational OSH culture underpinned by key OSH principles, and awareness/competence development on psychosocial risk management can have a positive effect on organizational action. However, further support needs to be provided to organizations around developing primary prevention interventions at the organizational level with the aim of reducing job demands.

## 1 Introduction

Psychosocial risks are those aspects related to work design, organization and management, and their social contexts that may have the potential to cause harm to workers' health and well-being [1]. Work-related stress, violence, harassment, and burnout are some of the main impacts associated with exposure to psychosocial risks at work [2]. Psychosocial risk management in the workplace has been one of the main concerns in occupational safety and health (OSH) in Europe over the last decades [3]. Nevertheless, this remains a challenge for organizations due to rapid changes in the world of work (e.g., technological development, digitalization, remote and hybrid work, and workforce diversity) which are associated with the exacerbation of existing risks, on one hand, and the emergence of new ones, on the other. The European Union Strategic Framework on OSH 2021-2027 calls for cooperation among member states and social partners to anticipate emerging risks linked to world changes, and psychosocial risks are recognized among the main priorities in the future of work [[4], [5], [6]]. This paper aims to explore whether key OSH policy principles are associated with organizational action on work-related stress, a better psychosocial work environment, and less reported stress in Italy, where specific legislation on work-related stress was introduced in 2008.

It has been acknowledged that legislation represents a strong driver for action in psychosocial risk management at both European and national levels [3,7]. The European Framework Directive 89/391/EEC on OSH established the obligation of employers to assess and manage all types of OSH risks—including psychosocial risks—in all member states [8]. This resulted in many EU member states to include prevention and management of psychosocial risks into their national-level OSH regulation [9] and evidence has shown the effective role of legislation in driving organizational action [10].

The Italian national policy context is particularly interesting because it presents one the earliest examples of specific legislation in this area, and is well established [11,12]. The Italian Legislative Decree 81/08 harmonized the OSH provisions of many previous regulations introduced over sixty years and furthermore made explicit the obligation of the employer to assess and manage the risks associated with work-related stress, in accordance with the contents of the 2004 European framework agreement on work-related stress. Following that, national guidelines on psychosocial risk management were developed by the Italian national OSH Consultative Permanent Committee. Furthermore, an evidence-based and integrated methodological approach was developed to support Italian organizations in the management of risks associated with work-related stress aligned to the national legal requirements [11]. This methodology is currently the most used by Italian organizations [12] and it is based on a participatory approach, starting from the identification of psychosocial hazards to the implementation of corrective and preventive actions.

Monitoring of national data from the European Survey of Enterprises on New & Emerging Risks (ESENER) in Italy highlighted a decrease from 2009 to 2014 in the concern reported by employers regarding the presence of psychosocial risks in organizations [13,14]. This was also associated to a strong improvement in actions and procedures to manage psychosocial risks for preventing work-related stress [15]. This highlighted the key role of legislation that was implemented between 2008 and 2010 as a driver for psychosocial risk management in organizations. Nevertheless, this trend showed a slowdown from 2014 to 2019 [16] and this raises a question about organizational effectiveness in translating policies into meaningful actions.

A recent paper published by Jain and colleagues [17] highlighted a significant association between country-specific national legislation on stress and the existence of organizational-level stress action plans and, in turn, an increase in reported job resources by the European workforce. However, job resources were only found to be associated with less reported work-related stress in those countries with specific legislation on work-related stress and psychosocial risks. Given the aforementioned evidence and policy and practice developments in Italy, this study aims at verifying these associations in the Italian context and draw inference on the effectiveness of the national legal framework.

There is an increasing interest in understanding the reasons underpinning the inconsistent effects of occupational health interventions to improve working conditions and employee health and well-being [18,19]. Organizational-level interventions aim to eliminate or reduce the sources of stress (primary-level interventions) in the work environment [20]. However, Jain and colleagues [17] argued that in countries where no specific legislation on psychosocial risks exists, actions put in place by organizations might be more oriented towards the development of individual resources and less towards the improvement of work organization and job design, and creating healthier psychosocial work environments. Indeed, primary-level interventions to deal with work-related stress can be perceived to be more complex and time-consuming to be effective [21], thus organizations might be more likely to introduce secondary and tertiary-level interventions aiming at the individual employee level. Moreover, there is evidence that the success of organizational-level interventions implies several further aspects related to their implementation process [18,22].

This study aims to partially test the findings from Jain et al. [17] focusing on Italy where the OSH legislative framework was updated in 2008 to include specifically the assessment and management of psychosocial risks. We extend the Jain et al. study by also focusing on the role of key OSH policy principles. Every key OSH framework legislation and indeed OSH management policy and system is based on some key principles. These include management commitment to OSH, management prioritization of OSH in business decision-making, organizational communication on OSH, and employee consultation and participation (e.g. 23). These principles are also reflected in the assessment of organizational OSH culture and climate. For example, the Psychosocial Safety Climate (PSC) theory [24], postulates four subsystems of PSC: management commitment to psychological health and safety, management prioritization of employee psychological health and safety, organizational communication, and organizational participation. Hence, the aim of this study is to explore the relationship between these key OSH principles with the implementation of organizational action plans to prevent work-related stress as reported by Italian employers, and whether these are associated with better psychosocial working conditions (in terms of job demands and resources on the basis of the Job Demands Resources model [25,26] and less reported work-related stress as reported by Italian workers. We therefore hypothesize the following.

H1 OSH policy principles are positively associated with organizational stress action plans.

H2 Organizational stress action plans are negatively associated with job demands.

H3 Organizational stress action plans are positively related to job resources.

H4 Organizational stress action plans are negatively related to work-related stress.

H5 Job demands are positively related to work-related stress.

H6 Job resources are negatively related to work-related stress.

## 2 Materials and methods

### 2.1 Data sources and participants

We used data for Italy from two different European-level surveys that each used a multistage stratified random sampling design. Table 1 presents the sample characteristics from both datasets. The first was the 2nd (employer) European Survey of Enterprises on New and Emerging Risks (ESENER-2) that was carried out in 2014 which records how health and safety is organized at workplaces across 36 European countries [14]. The survey encompasses public and private establishments with more than five employees, with “the person who knows best about health and safety in this establishment” through computer-assisted telephone interviewing. Data from 2254 Italian establishments was collected, although we only included enterprises which had at least 10 employees (N = 1656).

**Table 1:** Sample characteristics.

		ESENER	EWCS
Size	10-249	1422	344
	250+	234	150
Industry	Agriculture, forestry, fishing, mining, energy, and water	57	14
	Manufacturing	414	103
	Construction	124	21
	Distribution, hotels, and restaurants	248	64
	Transport and communication	133	50
	Banking and finance	158	50
	Public admin, education, and health	491	179
	Other services	31	13
Sex	Male	—	243
	Female	—	251
Contract	Full time	—	406
	Part time	—	88

The second data source was the (employee) 6th European Working Conditions Survey (EWCS) [27]. Based on face-to-face interviews in 2015 from 35 European countries, the survey covers a range of employment statuses, working conditions, and worker health to capture the multifaceted dimensions of work in Europe. For the present study, we included only Italian employees working in organizations with at least 10 employees resulting in 494 respondents (50.8% female; *M_age* = 46, SD = 9.8). The two datasets were chosen to allow for the analysis of the relationship between psychosocial risk management organizational practices reported through ESENER in 2014 and psychosocial working conditions and the experience of employee work-related stress as reported through the EWCS in 2015. Even though a more recent ESENER dataset exists, there is no recent EWCS complete dataset due to the Covid-19 pandemic.

### 2.2 Measures and data analysis

We replicated part of the analysis model in the Jain et al. study using the Italian data and adding OSH principles to the model. Table 2 presents a summary of the items used to develop the measures used. Data analysis was conducted using the R-statistical software version 4.3.1 [29], and two main R packages were employed: *lavaan* version 0.6-16 for structural equation modeling (SEM), and *tidyverse* version 2.0.0 for data manipulation. As in the Jain et al. study, we followed a four-stage analysis approach. First, we standardized all items in our datasets as indexes ranging from 0 to 100 to achieve equality in range and variance and reduce multicollinearity [30], and dichotomous responses were coded as “0 = No” and “100 = Yes”. Second, we carried out a confirmatory factor analysis on the job demands and job resources factors at the individual level from the sixth EWCS [28]. The categorical least squares (cat-ULSMV) estimation [31] procedure was used to fit the data to the proposed model which confirmed an acceptable fitting model (RMSEA = .08; SRMR = .08; CFI = .92; TLI = .90; *χ*^2^ = 770.8, *df* = 254, *p* < .001), and reliability coefficients (Cronbach's alpha between .67 to 90) [32]. The means of these items were then calculated to obtain an index score per person for job demands and job resources, as well as each respective sub-factor. The same procedure was used to evaluate the OSH policy principles model from ESENER-2 which achieved a good fit (RMSEA = 0; SRMR = .03; CFI = 1.0; TLI = 1.0; *χ*^2^ = 2.4, *df* = 5, *p* < .001), and reliability coefficient (Cronbach's alpha .77).

**Table 2:** Study measures.

Measure	Survey and items used
OSH policy principles	ESENER-2: 5 items: ‘*Are health and safety issues discussed at the top level of management*’, ‘*Do team leaders and line managers in your establishment receive any training*’, ‘*Do you regularly carry out workplace risk assessments*’, ‘*Do you have sufficient information on how to include psychosocial risks in risk assessments*’, ‘*Are health and safety issues regularly discussed in staff or team meetings*’
Organizational stress action plans	ESENER-2 - Single item: ‘*Do you have an action plan to prevent work-related stress*’
Job demands	6th EWCS - Latent factor comprising of three measures [informed by Eurofound research, 2019 [28]]: *Quantitative demands*, *emotional demands,* and *pace determinants*, each measured by three items
Job resources	6th EWCS - Latent factor comprising of four measures [informed by Eurofound research, 2019 [28]]: *Employee participation* (measured by three items), *job control* (measured by four items), *supervisor support* (measured by seven items), and (e.g., your immediate boss provides useful feedback on your work) and *support from colleagues* (measured by three items)

Work-related stress	6th EWCS - Single item: ‘*How often do you experience stress in your work*’

**Fig. 1:**
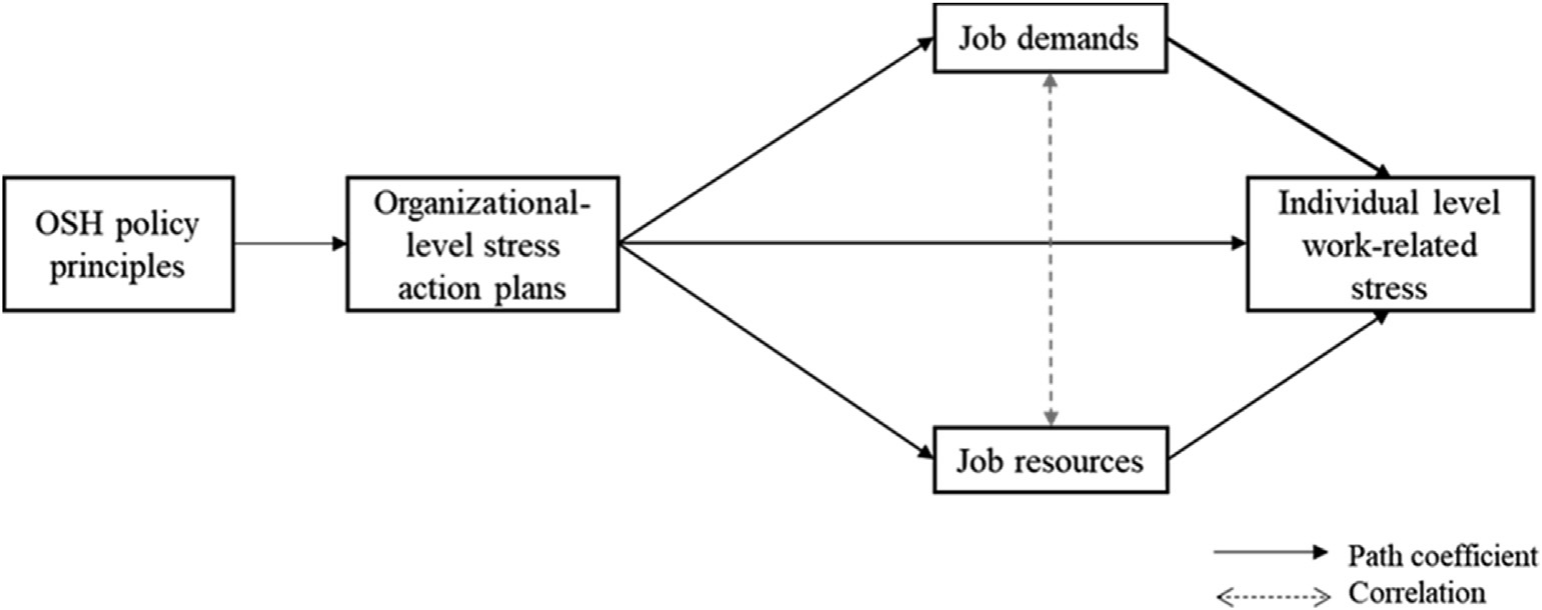
Research model.

Third, we linked the ESENER-2 and the 6th EWCS using the approach by Jain et al. as explained below. Responses from ESENER-2 were aggregated to the industry and company size level to create OSH policy principles and an organizational-level stress action plan index which were calculated considering the organization's size (10-249 and 250+ employees) within each specific industry. These indexes were assigned to each respondent of the sixth EWCS following the same criteria (industry and size). The result of this procedure is a consolidated database where each individual employee in the sixth EWCS has been assigned OSH policy principles and an organizational-level stress action plan index score calculated from the ESENER-2 which is specific to the company size and industry they belong to. Finally, a path analysis in SEM was fitted to the model proposed in Fig. 1. As data was moderately skewed, estimates were calculated using maximum likelihood with robust standard errors (MLR) [33]. We used bias-corrected bootstrapping (set at 1,000 at 95% confidence intervals) to simulate the sampling distribution of the coefficients. These tested the confidence intervals for the effects between OSH policy principles, work-related stress action plans, job demands, job resources, and individual-level work-related stress. As both indexes were calculated using the company size, we compare the results for both groups of companies (10-249 and 250+ employees).

## 3 Results

### 3.1 Correlations

Table 3 presents the descriptive statistics and correlations for the study measures. Size is significantly correlated to all variables in the analysis albeit less strongly with job demands. OSH policy principles are strongly correlated to organizational-level stress action plans and to a smaller extent with job demands and work-related stress. Organizational-level stress action plans are significantly correlated to job resources. Job demands are strongly correlated to work-related stress.

**Table 3:** Correlation matrix.

	Mean (SD)	1	2	3	4	5
1. OSH policy principles	78.4(2.6)					
2. Organizational-level stress action plans	57.5(12.0)	.66∗∗∗				
3. Work related stress	46.6(26.8)	.09∗	.03			
4. Job demands	35.2(20.1)	.11∗	.03	.38∗∗∗		
5. Job resources	61.3(18.3)	.02	.13∗∗	-.06	-.04	
6. Company Size^1^	—	.74∗∗∗	.64∗∗∗	.11∗∗	.10∗	.15∗∗

Notes: ^1^ 2 = 10-249; 3 = 250+. Below 10 employees are not included (=1).

∗p ≤ 0.05; ∗∗p ≤ 0.01; ∗∗∗p ≤ 0.001.

Table 4 presents differences between SMEs and large organizations in terms of OSH policy principles (*t = 24.92, df = 492, p < .001*), and organizational-level stress action plans (*t = 17.85, df = 492, p < .001*), which are both significantly different. As expected, large organizations are more likely to implement organizational-level stress action plans, but the findings also indicate that larger organizations also have higher adherence to OSH principles.

**Table 4:** Differences between SMEs and large organizations (ESENER-2 data)

	OSH policy principles [mean (SD)]	Organizational-level stress action plans [mean (SD)]	Sample (n)
10-249 employees	77.1 (1.9)	52.4 (5.8)	344
+250 employees	81.4 (1.4)	69.2 (15.1)	150

### 3.2 Testing the proposed model

**Fig. 2:**
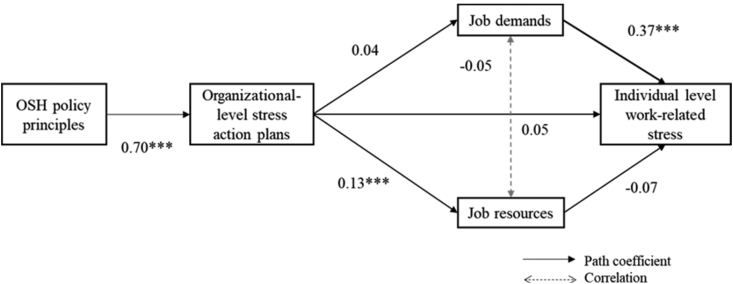
Standardised coefficients for the global model. Note: ∗p ≤ 0.05; ∗∗p ≤ 0.01; ∗∗∗p ≤ 0.001.

The model tested (Fig. 2) demonstrated an acceptable fit (RMSEA = .07; SRMR = .03; CFI = .98; TLI = .92; χ2 = 7.2, *df* = 3, *p* > .05). The path from OSH policy principles to action plan was highly significant (C.I. = .62-.78 *p* < .001) indicating that companies are more likely to implement action plans to manage stress, where there is stronger adherence to OSH policy principles, thereby confirming hypothesis 1. Hypothesis 2 (organizational stress action plans are negatively associated with job demands), and hypothesis 4 (organizational stress action plans are negatively related to work-related stress) were not confirmed as the path coefficients were not statistically significant. However, hypothesis 3 (organizational stress action plans are positively related to job resources) and hypothesis 5 (job demands are positively related to work-related stress) were confirmed as the path from action plans to job resources (C.I. = .02-.24, *p* < .05), and the path from job demands to individual-level work-related stress were significant (C.I. = .27-.47, *p* < .001). This implies that when companies have implemented action plans to manage stress, these are likely to focus on increasing job resources, and not decreasing job demands, even though these are associated with more reported work-related stress. Finally, even though organizational action plans are significantly associated with higher job resources, these are not significantly associated with less reported work-related stress. Therefore hypothesis 6 was rejected (job resources are negatively related to work-related stress).

## 4 Discussion and conclusions

Recently, Jain and colleagues [17] highlighted that the existence of national-level legislation is associated with more organizations putting in place action plans to prevent work-related stress which were associated with providing more job resources but did not result in reducing job demands. Furthermore, job resources were only found to be associated with less reported work-related stress by the European workforce in those countries with specific legislation on work-related stress and psychosocial risks, like Italy. The current study explored the applicability of these findings with a focus on Italy which introduced specific legislation and practices to deal with work-related stress in 2008 [15]. It extends the Jain et al. study by exploring the role of key OSH policy principles as a motivator of organizational action on work-related stress. OSH legislation, including Italian Legislative Decree 81/08, is underpinned by the key principles of management commitment to OSH, management prioritization of OSH in business decision-making, organizational communication on OSH, and employee consultation and participation [23]. Furthermore, since the update of Decree 81/08 Italian employers are specifically required to assess and manage work-related stress risks, and tools have been made freely available to organizations to enable them to do so.

Our findings partly corroborated what emerged in the Jain et al. [17] study. No significant association was found between organizational action plans and a reduction in job demands. The existence of an organizational action plan was significantly associated with the provision of more job resources however, contrary to the Jain et al. study, these were not found to be associated with less reported work-related stress by the Italian workforce which raises concerns about the nature of resources provided. Similar concerns about organizational effectiveness in translating policies into meaningful actions were raised in an earlier study using the ESENER Italian dataset [16].

Our study lends strong support to the relationship between key OSH policy principles, which are embedded in Italian legislation, and organizational action on work-related stress. In this sense, legislation promotes an organizational OSH climate based on these key principles which drives organizations to put in place actions to deal with work-related stress. These principles are also reflected in theories and tools such as Psychosocial Safety Climate (PSC) which can be useful for the measurement of these principles in practice and can thereby contribute to the evaluation of policy initiatives on psychosocial risks at work. However, our findings highlight that while legislation and OSH principles are strong motivators for organizational action, this does not seem to be sufficient to orientate organizations towards effective interventions and implementing a preventive approach to address psychosocial risks and work-related stress.

Despite the mature national policy context in Italy where there is specific legislation, tools, and guidance, organizations seem to be more inclined to implement secondary and tertiary-level interventions, focused on improving employee skills to manage their work stress and on rehabilitation when stress has negatively impacted their health and work attendance, rather than primary-level interventions which deal with the sources of work-related stress in terms of work organization and design, and the creation of healthy psychosocial work environments [3,34]. Organizations often perceive primary-level interventions to be more complex or time-consuming [21], or they may lack competencies to effectively translate risk assessment results into appropriate organizational-level actions [18,22].

As a minimum legal requirement, Italian organizations must conduct a risk assessment including all psychosocial risk factors associated with the emergence of work-related stress, using a participatory approach [35]. Findings from the risk assessment must then be translated into actions aiming to eliminate or reduce the sources of work-related stress or to protect the workers who are exposed. Nevertheless, actions and interventions put in place often do not seem to achieve the desired outcomes [19,36]. The success of organizational-level interventions implies different key aspects that must be considered in the implementation process such as a valid risk assessment, employee participation, communication and motivation to support the intervention, and line managers' support or readiness for change [18,22]. It is possible that some of these competencies are lacking in Italian organizations [36]. Previous studies have shown very low involvement of an in-house or externally contracted psychologist in health and safety services in Italy [15]. The involvement of specialist expertise—particularly when not already available in organizations—may be helpful in putting in place effective and appropriate interventions to tackle work-related stress. To avoid inconsistent and ineffective organizational action plans there is a growing need for developing competencies and expertise of those having an active role in the management of psychosocial risks [13], who are recognized in Italian legislation as employers, supervisors, health and safety representatives, occupational physicians and workers' representatives. Thus, initiatives at policy level should place emphasis on improving knowledge and skills in organizational OSH services. Furthermore, some evidence does exist on the inconsistent effects of organizational-level interventions, even when they are implemented under evidence-based methodologies and theoretical frameworks [18]. Among others, it may be that the interventions fail because they are not really oriented towards improving the outcomes that should be improved [18,19]. This again raises the question of what kind of resources are targeted through organizational-level actions in Italy.

Finally, Italian policies only focus on work-related stress as this specific term is included into the Italian legal framework on OSH stating the obligation to assess the risks associated with it. Although OSH legislation is underpinned by the control cycle framework in order to identify and eliminate or reduce risks to workers' health, safety, and well-being and avoid harm, it is important that further efforts are made towards educating organizations about the positive benefits of managing psychosocial risks in terms of employee engagement, motivation, and job satisfaction. This could result in more organizational investment in effective organizational-level interventions aiming at developing psychosocially healthy work environments, and not only at individual resources and the ability to cope with stress.

This study presents some important limitations that must be acknowledged. First, data are cross-sectional and self-reported. This limits drawing conclusions about causality and controlling the possibility of common method variance in the findings [37]. Future studies should consider a longitudinal design, even if this could affect sample size. This study used data from the 6th EWCS and the 2nd ESENER to offer an in-depth exploration of the Italian national data and verify the findings of the European-level Jain et al. study. Verifiability is a core principle of science and is important as the generation of new knowledge [38]. Large supranational surveys on working conditions offer a broad comprehensive source of information, allow for comparisons across countries, and can develop evidence-based knowledge by providing useful insights for policy and practice. Nevertheless, it is important to also consider findings from national surveys where available, since these are more contextualized into the national regulatory framework and main priorities at national level, and might allow the identification of prevention strategies tailored to the needs of national OSH systems. Furthermore, national-level surveys might present the opportunity to explore the views of larger and more representative samples. Future studies should consider replicating similar models using data from Italian national surveys, in order to further explore and confirm the current findings.

## Conflicts of interest

All authors have no conflicts of interest to declare.
